# Bifunctionalized Silver Nanoparticles as Hg^2+^ Plasmonic Sensor in Water: Synthesis, Characterizations, and Ecosafety

**DOI:** 10.3390/nano9101353

**Published:** 2019-09-20

**Authors:** Paolo Prosposito, Luca Burratti, Arianna Bellingeri, Giuseppe Protano, Claudia Faleri, Ilaria Corsi, Chiara Battocchio, Giovanna Iucci, Luca Tortora, Valeria Secchi, Stefano Franchi, Iole Venditti

**Affiliations:** 1 Department of Industrial Engineering and INSTM, University of Rome Tor Vergata, via del Politecnico 1, 00133 Rome, Italy; paolo.prosposito@uniroma2.it (P.P.); luca.burratti@uniroma2.it (L.B.); 2 Center for Regenerative Medicine, University of Rome Tor Vergata, Via Montpellier 1, 00133 Rome, Italy; 3 Department of Physical, Earth and Environmental Sciences, University of Siena, Via Mattioli 4, 53100 Siena, Italy; arianna.bellingeri@student.unisi.it (A.B.); giuseppe.protano@unisi.it (G.P.); ilaria.corsi@unisi.it (I.C.); 4 Department of Life Sciences, via Mattioli 4, 53100 Siena, Italy; faleric@unisi.it; 5 Department of Sciences, Roma Tre University of Rome, Via della Vasca Navale 79, 00146 Rome, Italy; chiara.battocchio@uniroma3.it (C.B.); giovanna.iucci@uniroma3.it (G.I.); luca.tortora@uniroma3.it (L.T.); valeria.secchi@uniroma3.it (V.S.); 6 Surface Analysis Laboratory INFN Roma Tre, via della Vasca Navale 84, 00146 Rome, Italy; 7 Elettra-Sincrotrone Trieste S.c.p.A., Strada Statale 14, km 163.5, 34149 Basovizza Trieste, Italy; stefano.franchi@elettra.eu

**Keywords:** silver nanoparticles, Hg^2+^ sensors, heavy metal sensing, plasmonic sensors, optical sensors, ecosafety

## Abstract

In this work, hydrophilic silver nanoparticles (AgNPs), bifunctionalized with citrate (Cit) and L-cysteine (L-cys), were synthesized. The typical local surface plasmon resonance (LSPR) at λ _max_ = 400 nm together with Dynamic Light Scattering (DLS) measurements (<2R_H_> = 8 ± 1 nm) and TEM studies (Ø = 5 ± 2 nm) confirmed the system nanodimension and the stability in water. Molecular and electronic structures of AgNPs were investigated by FTIR, SR-XPS, and NEXAFS techniques. We tested the system as plasmonic sensor in water with 16 different metal ions, finding sensitivity to Hg^2+^ in the range 1–10 ppm. After this first screening, the molecular and electronic structure of the AgNPs-Hg^2+^ conjugated system was deeply investigated by SR-XPS. Moreover, in view of AgNPs application as sensors in real water systems, environmental safety assessment (ecosafety) was performed by using standardized ecotoxicity bioassay as algal growth inhibition tests (OECD 201, ISO 10253:2006), coupled with determination of Ag^+^ release from the nanoparticles in fresh and marine aqueous exposure media, by means of ICP-MS. These latest studies confirmed low toxicity and low Ag^+^ release. Therefore, these ecosafe AgNPs demonstrate a great potential in selective detection of environmental Hg^2+^, which may attract a great interest for several biological research fields.

## 1. Introduction

Nanosized inorganic particles, in simple or composite formulation, have unique physical and chemical properties and represent an increasingly important material in the development of novel nanodevices that can be used in numerous fields such as catalysis, energy, optoelectronics, biomedicine, and sensors [1,2,3,4,5,6,7,8,9,10,11]. Several recent achievements show the opportunity of producing new types of nanostructured materials with planned surface and desired physico-chemical properties [12,13,14,15,16]. Among other materials, silver nanoparticles (AgNPs) are deeply studied for their optical and antibacterial properties, easy functionalizations and cheap preparations [17,18].

Nowadays, for the AgNPs use in sensing field, the main goal is the preparation of uniform nanosized particles with precise requirements in terms of size, shape, surface functionalities, and structural features [19,20,21,22]. In fact, many studies involved AgNPs as optical sensor, using the localized surface plasmon resonance (LSPR) that is specific feature of the colloidal silver nanoparticle solutions: the energy of the absorption maximum and the shape of the peak are strongly related with the size, shape and interparticle distance, but also with the surrounding environment, which can influence the degradation or aggregation of the particles [23,24,25]. These optical properties of NPs have allowed researchers to mature new diagnostic methods that are useful for optical and colorimetric measurements [26,27,28].

In particular, AgNPs have drawn great attention for mercury sensors development, especially due to their high sensitivity to Hg^2+^ micro-level concentration changes [29,30]. This behavior has been related, both with redox chemistry of Hg^2+^ and silver surface (Ag°), and both with the soft-soft chemistry between metals and sulfur-containing capping agents used for AgNPs [31]. In both cases, the mercury presence produce important changes in the absorbance intensity and peak position of AgNPs. Surface functionalization has the main role both in stabilization and in reactivity of AgNPs. Therefore, the ligand surface chemistry is used to tune their sensing properties. For example, many ligands, such as *N*-cholyl-Lcysteine [32], citrate [33,34,35], 6-thioguanine [36], cytosine triphosphate [37], have been used for the surface modifications of AgNPs and AuNPs, and the different functionalizations change the inter and intra-ligand interactions. Obviously, all this produces different properties and capacities of the nanoparticles, that can aggregate or disaggregate and interact with elements and ions present in their surroundings, and it affects sensing features towards metals ions, such as Hg^2+^, modifying the selectivity and sensitivity. So far there are several reports available for the colorimetric detection of Hg^2+^ ions using green synthesized unmodified AgNPs in aqueous medium [38]. Frequently used, citrus extracts from lemons and sweet orange fruits have been investigated for green synthesis of AuNPs and AgNPs used as Hg^2+^ colorimetric sensors [39]. There are many bio extracts reported from different plants, fruits, leafs, etc. which have been used for photoinduced green synthesis of AgNPs where a bio extract acts as reducing as well as stabilizing agent and applied in Hg^2+^ detection [38,39,40].

Nevertheless, the use of NPs for heavy metals removal known as nanoremediation is a matter of concern due to the potential risks associated with the scarcity of information regarding their behavior, fate and impact on the environment and human health [41]. In order to overcome such limitations, an ecosafe approach is developed with the aim to test the ecotoxicity of NPs to selected biota having an important ecological role in aquatic ecosystems, as for instance primary producers such as freshwater and marine microalgae [42,43].

AgNPs are recognized to exert toxic effects to biota, both from fresh- and marine waters, from bacteria [44] and microalgae [45,46,47,48] to crustacean [49], mussels [50], fishes [51] and mammalian cells [52,53]. Among these studies, those reporting Ag^+^ release in exposure media [44,45,46,47,48,52,54], report that AgNPs toxicity is often closely linked to the dissolution of the particles and consequent release of Ag ions during exposure. Therefore, based on such evidence, the ecosafety assessment of AgNPs is mandatory in order to avoid any potential toxicological risks associated with their application in environmental remediation.

In this work AgNPs functionalized with hydrophilic capping agents, i.e., citric acid (Cit) and L-Cysteine (L-cys) were synthesized and characterized by means of UV-Vis, Fourier Transform Infrared (FTIR), Synchrotron radiation-X-ray photoelectron (SR-XPS), Near Edge X-ray Absorption Fine Structure (NEXAFS) spectroscopies. Their nanodimensions were confirmed by Dynamic Light Scattering (DLS) and Transmission Electron Microscope (TEM) analysis. AgNPs were tested as plasmonic sensor for heavy metal detection in water, showing selectivity and sensitivity for Hg^2+^ ions. The AgNPs-Hg^2+^ system was deeply studied by means of UV-Vis, and, SR-XPS spectroscopies, DLS, Inductively Coupled Plasma-Mass Spectrometry (ICP-MS) and TEM studies. Moreover, in view of AgNPs application in real water systems, their ecosafety was investigated.

## 2. Experimental

### 2.1. Materials and Methods

Sodium citrate (Na_3_C_6_H_5_O_7_, Cit), L-Cysteine (C_3_H_7_NO_2_S, L-cys), silver nitrate (AgNO_3_) and sodium borohydride (NaBH_4_) have been used as received (reagent grade, Sigma-Aldrich, St. Louis, MO, USA). Metal ions contamination was accomplished by using the following salts:

NaAsO_2_, NaHAsO_4_·7H_2_O, Ca(ClO_4_)_2_, Cd(NO_3_)_2_, CoCl_2_·6H_2_O, CrCl_3_·6H_2_O, Cu(NO_3_)_2_, FeCl_3_·6H_2_O, Hg(NO_3_)_2_·H_2_O, KClO_4_, Mg(ClO_4_)_2_, NaClO_4_, NdCl_3_·6H_2_O, NiCl_2_·6H_2_O, Pb(NO_3_)_2_, Zn(NO_3_)_2_·6H_2_O. For all the solutions, we used deionized water (electrical conductivity less than 1 µΩ/cm at room temperature) obtained from Millipore Milli-Q water purification system. All the reagents were purchased (from Sigma-Aldrich, St. Louis, MO, USA) and were used without further purification.

UV-Vis spectra were run in H_2_O solution by using quartz cells with a Shimadzu 2401 PC UV-vis spectrophotometer and by using single-use UV-PMMA cuvettes with Perkin-Elmer Lambda 19 UV-Vis-NIR for sensing test characterization. ATR-FTIR spectra have been recorded for films deposited by casting from water suspension with an FTIR spectrometer (Nicolet iS50, Thermo Fisher Scientific, Madison, WI, USA) equipped with a mid- and far-IR capable diamond ATR accessory. FT-IR spectra were recorded in the range between 350 and 4000 cm^−1^ with a resolution of 4 cm^−1^, a zero-filling factor of 2 and the co-addition of 64 scans. Data were acquired using OMNIC software (version 9.8.372, Thermo Scientific), subtracting the air background spectrum obtained prior to each sample spectrum acquisition. AgNPs were investigated by TEM (Philips Morgagni 268 D electronics, at 80 KV and equipped with a MegaView II CCD camera, Philips Electronics, Eindhoven, The Netherlands) at 10 mg/L in MilliQ water. The obtained images were analysed with *ImageJ* for particles size measurement. A total of 180 particles were measured in two portions of two different images and the average size was calculated. Size distribution of AgNPs (50 mg/L) have been investigated by means of DLS (Zetasizer Nano Series, Malvern instruments, Enigma Business Park, Grovewood Rd, UK), combined with the Zetasizer Nano Series software (version 7.02, Particular Sciences) at T = 25.0 ± 0.2 °C in milliQ water, as well as media used for toxicity assessment (freshwater, TG 201, and marine water, F/2). Correlation data have been acquired and fitted in analogy to our previous work [55,56]. Ag^+^ release from the AgNPs has been assessed in both TG 201 and F/2 aqueous media, at 0 h and after 72 h. Six solutions have been prepared: TG 201, F/2, TG 201 + 500 µg AgNPs/L, F/2 + 500 µg AgNPs/L, TG 201 + 7 µg AgNO_3_/L, F/2 + 7 µg AgNO_3_/L. Solutions were kept in the same conditions as toxicity tests (22 ± 2 °C and 16/8 light-dark photoperiod) and mixed by shaking once a day. An aliquot of each solution was taken at 0 h and after 72 h and centrifuged (5000 g, 40 min, 22 °C) by using a centrifugal filter device with a 3 kDa cut-off (Amicon Ultra-15 mL, Millipore, Sigma-Aldrich, St. Louis, MO, USA). The resulting filtrate was acidified with HNO_3_ (10%) and analysed by ICP-MS (Perkin Elmer NexION 350 spectrometer) for determining Ag concentration.

SR-XPS measurements were performed at the Elettra synchrotron radiation source (Trieste, Italy), using the Materials Science Beamline (MSB), that is positioned at the left end of the bending magnet 6.1. MSB is equipped with a plane grating monochromator providing SR light in the 21–1000 eV energy range. The UHV endstation, whose base pressure is of 2 × 10^−10^ mbar, is equipped with a SPECS PHOIBOS 150 hemispherical electron analyser, low-energy electron diffraction optics, a dual-anode Mg/Al X-ray source, an ion gun, a sample manipulator with a K-type thermocouple attached to the rear side of the sample. For the here presented experiments we detected photoelectrons emitted by C1s, O1s, S2p, Ag3d, N1s and Hg4f core levels, using a normal emission geometry. In order to maximize signals intensity, we selected a photon energy value of 630 eV (impinging at 60°) for all elements except S; in order to maximize the intensity of S2p signals, that was expected to be very low due to element dilution, the S2p core level was measured with photon energy = 350 eV. Charging correction of binding energies (BEs) was done using as a reference the aliphatic C1s (BE 285.0 eV) [57]. To fit core level spectra, we subtracted a Shirley background and then used Gaussian peak functions as signals components [58,59].

NEXAFS experiments were carried out at the BEAR (Bending magnet for Emission Absorption and Reflectivity) beamline, installed at the left exit of the 8.1 bending magnet and located the ELETTRA third generation storage ring. The beamline optics can deliver photons having energy comprised between 5 eV and 1600 eV with selectable degree of ellipticity. The carbon K-edge spectra were recorded at normal (90°) and grazing (20°) incidence relative to the sample surface of the linearly polarized photon beam; however, no angular effects were detected. Calibration of the photon energy and resolution was carried out at the K absorption edges of Ar, N_2_ and Ne. In order to normalize the spectra, a straight line fitting the part of the spectrum below the edge was subtracted and the value recorded at 330.00 eV was assessed to 1.

### 2.2. AgNPs Synthesis

The AgNPs stabilized with citrate (Cit) and L-Cysteine (L-cys) were prepared and characterized in analogy to literature reports [60,61]. 1.47 g of sodium citrate were dissolved in 50 mL of distilled water (0.01 M), 0.006 g of L-cys in 25 mL of distilled water (0.002 M) and 0.21 g of AgNO_3_ in 25 mL of distilled water (0.05 M). Then, 25 mL of L-cys solution, 10 mL of Cit solution and 2.5 mL of AgNO_3_ solution were added sequentially in a 100 mL flask, provided with a magnetic stir. The mixture was degassed with Argon for 10 min, then 4 mL of NaBH_4_ solution (0.016 g in 4 mL distilled water) were added. The mixture was allowed to react at room temperature for 2 h and at the end the brown solution was recollected and purified by centrifugation (13,000 rpm, 10 min, 2 times with deionized water). AgNPs main characterizations: UV-Vis (λ_max_ [nm], H_2_O) = 400 nm; < 2R_H_ > ([nm], H_2_O) = 8 ± 1; TEM Ø = 5 ± 2 nm.

### 2.3. AgNPs Sensing Tests

A fixed volume of AgNPs in water (typical concentration 1.6 mg/mL) was mixed with a fixed volume of water solution containing the heavy metal ions at specific concentration. After five minutes of interaction of the nanoparticles with the metal ions, the optical absorption spectra were collected in order to verify possible changes (shape, energy and intensity) of the typical localized surface plasmon resonance (LSPR). The response to several metal ions at different concentrations from 25 ppm down to 1 ppm was tested by UV-Vis spectroscopy [27].

### 2.4. AgNPs Ecosafety Tests

The intrinsic toxicity of AgNPs was investigated through standardized 72 h algal growth inhibition tests [62,63] using two different algal species, belonging to the freshwater and to the marine water environment, *Raphidocelis subcapitata* and *Phaeodactylum tricornutum*. Algae were cultured, respectively, in TG 201 medium and F/2 medium, in axenic and exponential growth conditions, at 18 ± 1 °C and 16/8 h light-dark cycle photoperiod. In order to reduce the introduction of chelating agents which have been shown to interfere with heavy metals toxicity [64,65], algal toxicity tests were run with the same medium used for algal culturing modified only in the concentration of ethylenediaminetetraacetic acid (EDTA) (0.05 mg/L and 0.8 mg/L respectively as previously proved to ensure acceptable algal growth). PS single-use sterile multiwell were used for toxicity tests, with 2 mL volume capacity for each well. Algae were acclimated 72 h before running the test under the following conditions: 22 ± 2 °C and 16/8 light-dark photoperiod. Initial algal concentration was 1 × 10^4^ cells/mL and tests were manually aerated every 24 h using a pipette with sterile tips. Exposure concentrations were as follows: 10, 25, 50, 100, 200, 500 µg AgNPs/L. AgNO_3_ was used as positive control at the following concentrations of 3.5, 7, 14, 21, 35 µg/L. In order to exclude any possible role of coating components the toxicity of the AgNPs, citrate and L-Cysteine were tested separately at the following concentrations of 0.5, 1, 2, 5, 10 mg/L.

## 3. Results and Discussion

### 3.1. AgNPs Synthesis and Characterizations

The synthesis of AgNPs by wet chemical reduction is a useful method to obtain spherical nanoparticles with tuned sizes and opportune capping agent [60]. In this work, AgNPs have been prepared by the reduction in aqueous solution of silver nitrate with sodium borohydride as strong reducing agent. Two different capping agents have been chosen: Cit, crucial to induce a high hydrophilic behavior, and L-cys, to induce selective interaction with the environment.

**Figure 1:**
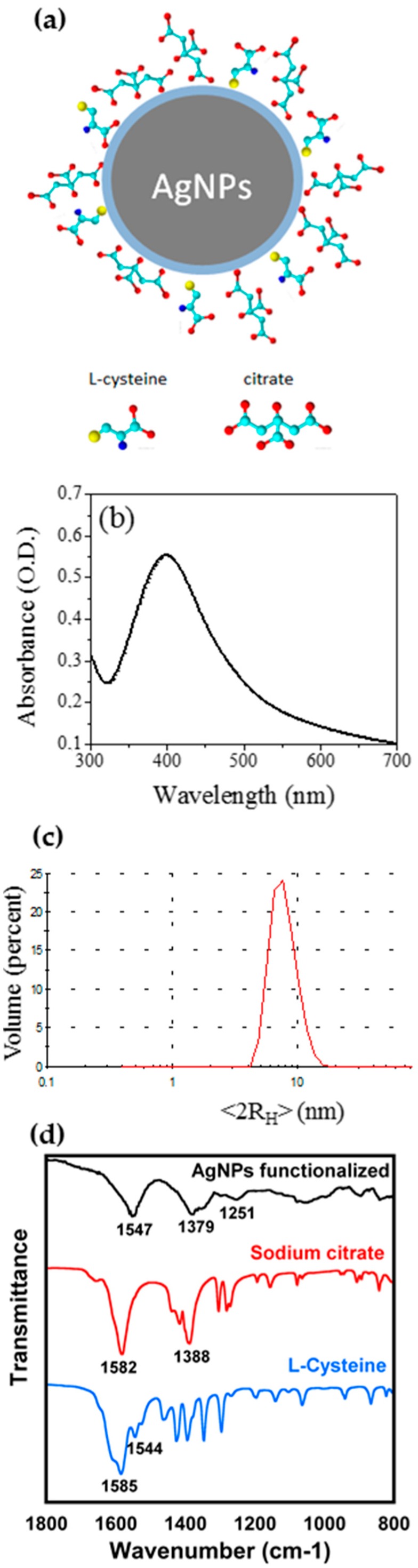
(**a**) Scheme of bifunctionalized hydrophilic silver nanoparticles (AgNPs); (**b**) UV-Vis spectrum in water of AgNPs, with local surface plasmon resonance (LSPR) band centred at 400 nm; (**c**) DLS measurements in water: <2RH> = 8 ± 1 nm; (**d**) ATR-FTIR spectra of bifunctionalized AgNPs (Top) and the capping agents, Cit (Center), and L-cys (Bottom).

In fact, it is well known that the amino group can easily interact with the chemical environment and in particular with Hg^2+^ ions [10]. In order to avoid the excessive presence of L-cys, leading to nanoparticles aggregation, the molar ratio Au/Cit/L-cys = 1/4/2 was chosen. The obtained AgNPs scheme is shown in Figure 1a. After careful purification, AgNPs have been characterized by means of UV-Vis, FTIR and XPS spectroscopies, by DLS and TEM studies. UV-Vis spectra have been carried out together with DLS measurements in water, as reported in Figure 1b,c. The UV-Vis spectrum showed the typical LSPR band, at λ _max_ = 400 nm, confirming the nanodimensions. DLS measurements in water showed hydrodynamic diameter <2R_H_> = 8 ± 1 nm, as expected. Moreover, FTIR investigations showed the presence of Cit and L-cys on the particles, as reported in Figure 1d.

When the ATR-FTIR spectrum of AgNPs bifunctionalized with Cit and L-cys was compared with reference spectra of these capping agents Figure 1d, it is immediately evident the absence of the band at ~1582 cm^−1^ (ν_as_(COO^−^)) in AgNPs spectrum [66]. This could be interpreted as evidence that most carboxylate groups of the citrate and cysteine are attached to the surface of the AgNPs. In fact, similar results was observed by Frost et al. [67], who studied the ATR-FTIR spectra of citrate-capped AgNPs and Au-NPs; only for AgNPs, they observed the disappearance of the ν_as_(COO^−^) peak and interpreted this result in terms of adsorption geometry of the citrate molecule, with all the three carboxylate groups interacting with the AgNPs surface: the ν_as_(COO^−^) lying parallel to the AgNPs surfaces results in an infrared-inactive transition. Moreover, in the AgNPs spectrum is present a broad peak at ~1547 cm^−1^ that could be due to the overlapping between the N-H bending of cysteine and the residual carboxylate groups not attached to the AgNPs surface. The functionalization with Cit and L-cys can be also supported by the presence in the AgNPs spectrum of a broad peak centered at 1379 cm^−1^ indicating the COO^−^ symmetric stretching vibrations; the peak position is slightly shifted to lower wavenumber compared to the free carboxylate anion, as already evidenced by Frost et al. [68]. The main contributions to the symmetric and asymmetric stretching vibration of the carboxylate group are obviously mainly due to the citrate molecule, that is present in higher concentration on the AgNPs; however, it seems reasonable to hypothesize a similar reactivity for the different carboxylate groups. The broad peak centered at 3300 cm^−1^ is attributed to the stretching vibrations of the hydroxyl group (ν(O–H)) (data not shown) probably coming from water adsorbed onto nanoparticles surface. We were not able to detect any S-H band around 2550 cm^−1^, that would indicate the presence of free L-cys on the AgNPs surface [68,69]. The intensity of this peak in the spectrum of L-cys is actually rather low (see Figure S2 in Supplementary Material), however, XPS results confirm the absence of S-H groups.

**Figure 2:**
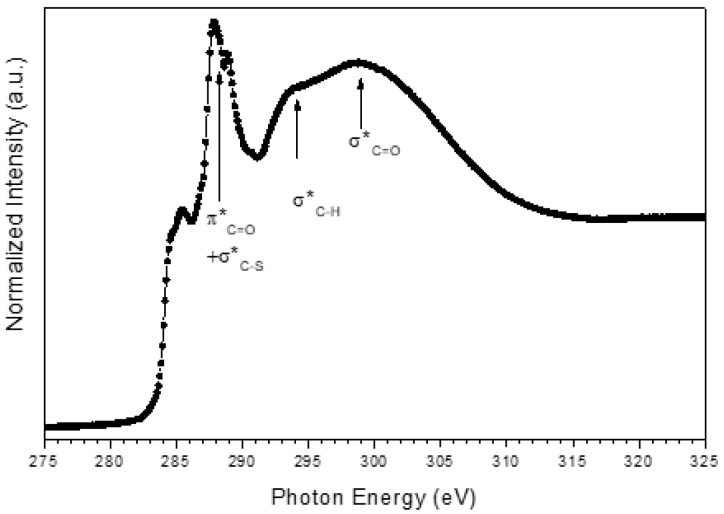
C k-edge NEXAFS spectrum of AgNPS recorded at 20° incidence angle.

To support FTIR data, AgNPs were also investigated by NEXAFS spectroscopy. C k edge spectra of AgNPs were recorded at normal, magic and grazing incidence; however, no angle-dependent effect was detected; therefore, the C k-edge spectrum of AgNPs is shown in Figure 2. The main feature in the spectrum is the 1s→π* consisting of two peaks located at 287.7 and 288.8 eV, with a shoulder at 285.5 eV. According to literature, the 1s→π* transition related to the C=O bond in the carboxylate of L-cys is expected at 288.6 eV [70]; similar values are expected for carboxyl groups [71]. Moreover, L-cys is expected to show a 1s→σ* peak related to the C-S bond at about 287.3 eV. Therefore, we can assign this complex band to overlap between π*C=O and σ*C–S transitions. The broad bands located at about 294 and 300 eV are related to 1s→σ* transitions of C–H and C=O bonds respectively [72]. The overall spectrum yields prove of successful capping of the AgNPs surface by both Cit and L-cys.

Moreover, the TEM studies carried out on diluted samples show AgNPs with narrow size distribution (TEM Ø 5 ± 2 nm) but with a certain tendency to aggregation, which could be due only to drying (see Figure S1). DLS measurements show a monodisperse population of NPs in solution These data are in agreement with DLS data. It can be noticed that the dimensions obtained from DLS are greater than those obtained from TEM images, since DLS estimated the hydrodynamic radius < 2R_H_ > of the particles in the aqueous environment and it is the Z-average value, which is the mean diameter weighted over the scattered light intensity. Therefore, the DLS data are not directly comparable with dimensions obtained from TEM images, as reported in the literature [7,56]. Anyway, the data confirmed in both cases the nanodimension of the AgNPs.

Measurements of Ag concentration in fresh and marine aqueous media showed an almost absent Ag^+^ ions release from AgNPs (Table 1) as opposed to what observed in the literature for other types of AgNPs [45,46,47,48,49,53,55].

In fact, Ag^+^ concentrations in both TG 201 medium (freshwater) and F/2 medium (marine water) with 500 µg AgNPs/L were low (up to 0.4 µg/L) and comparable with those measured in controls (up to 0.36 µg/L; Table 1). However, it is to be noted that in the presence of AgNPs, Ag^+^ levels slightly increased after 72 h, from 0.19 to 0.4 µg/L in freshwater, and 0.15 to 0.37 µg/L in marine water. Since AgNPs dissolution was demonstrated to be independent from particles aggregation state [73], the reason for the lack of ion release is probably to be found in the Cit/L-cys coating.

### 3.2. AgNPs Sensing Tests

**Figure 3:**
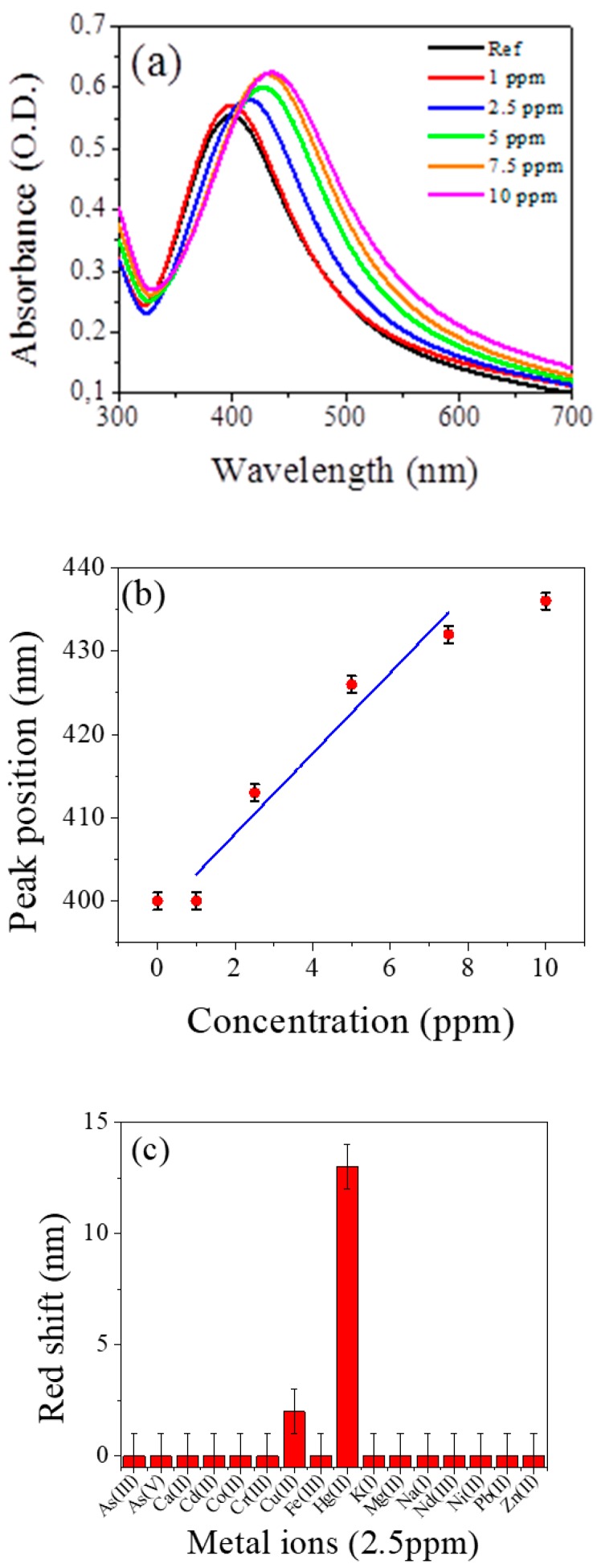
(**a**) Absorption spectra of AgNPs water solutions at room temperature and pH = 6.5 without and with different concentrations of Hg^2+^ listed in the figure; (**b**) calibration curve as a function of Hg^2+^ concentration; (**c**) redshift of the absorption band maximum in presence of all the metal ions tested at concentration of 2.5 ppm.

Sensing tests were made by checking the optical absorption spectra of the contaminated systems with respect to the reference AgNPs water solution (see also Figure S3). In Figure 3a the absorption spectra of AgNPs in water solutions without (reference solution) and with different concentrations of Hg^2+^ ions in the range 1 to 10 ppm, are reported. An evident red shift of the band peak energy together with an increase of the intensity and a broadening of the band with the increasing concentration of ions can be appreciated. The same type of measurement has been performed for all the 16 ions listed in the experimental part for concentration from 10 ppm down to the lowest concentration of 1 ppm. Figure 3b shows the red shift of the absorption band of all the metal ions tested at the concentration of 2.5 ppm. A significant change has been detected only for Hg^2+^ ions while for all the other ions the absorption bands remain almost constant within the error bars.

The limit of detection (LOD) of the nanosensor for the analysis of Hg^2+^ was determined using calibration curves. The LOD can be estimated as three times the standard deviation of the blank signal, obtaining a value equal to 0.6 ppm. The obtained LOD was found to be comparable with other AgNPs systems and the experimental results in the determination of Hg^2+^ obtained by some other methods are listed in Table 2 for comparison [8,10,40,74,75,76,77].

### 3.3. SR-XPS Characterization of AgNPs and AgNPs-Hg^2+^

**Figure 4:**
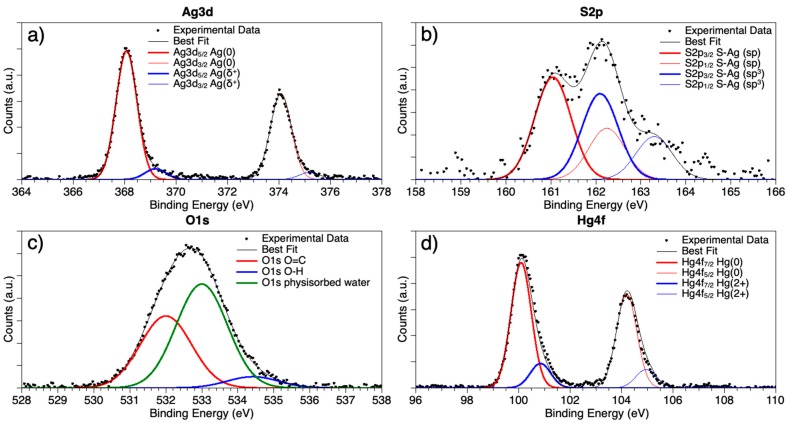
SR-XPS spectra collected on AgNPs-Hg^2+^ aggregates at (**a**) Ag3d; (**b**) S2p; (**c**) O1s and (**d**) Hg4f core levels.

Synchrotron radiation-induced X-ray photoelectron spectroscopy (SR-XPS) measurements allowed to probe the interaction between AgNPs and Hg^2+^ ions. Spectra were collected at C1s, N1s, O1s, Ag3d, S2p and Hg4f core levels (all BE (eV), FWHM (eV), relative intensity values and proposed signals assignments are reported in Supplementary Materials file, Table S1). All the individuated spectral components confirm AgNPs stability, and Cit and L-cys capping efficiency. C1s spectrum has three components at 285.00, 286.46 and 288.45 eV BE, respectively associated with aliphatic carbons (mainly impurities, that are always observed in samples prepared in air by liquid solutions); O1s spectra, reported in Figure 4c, also show three different kinds of oxygen atoms, respectively belonging to carbonyl (C=O) functional groups (532.00 eV BE), hydroxyl moieties (-OH, 533.00 eV BE) and physisorbed water (small contribution at about 534.5 eV BE). The atomic ratio between C=O and –OH is C=O/–OH = 1/1.4, very close to the C=O/–OH = 1/1.3 that is theoretically expected for a Cit/L-cys stoichiometry = 2/1, as in the synthetic procedure reported in Materials and Methods. N1s spectrum was also collected, showing a single component centred at 400.24 eV, as expected for amine-like nitrogens. Indeed, C1s, O1s, and N1s data analysis confirm the molecular stability of the AgNPs, also after interaction with Hg^2+^ ions.

Generally speaking, the most indicative signals for the surface-structure analysis of metal nanoparticles capped with thiols are M (Au4f, Ag3d) and S2p core levels; for this purpose, Ag3d and S2p spectra are reported in Figure 4 and will be here discussed in detail.

Ag3d spectra Figure 4a are asymmetric at high BE, a common feature in capped nanoparticles [20,27], indicating that at least two different kinds of silver atoms compose the nanoparticle: the spin-orbit pair at lower BE values (Ag3d5/2 = 368.08 eV) is associated with metallic silver at the NPs core; the signal at higher BE, of very small intensity (about 9% of the whole signal) is due to positively charged silver atoms at the NP surface, interacting with the capping molecule [20,27]. S2p spectra are also composite, showing two spin orbit pairs of very similar intensity (atomic percents are 54.4% lower BE–45.6% higher BE, as reported in Table S1 in the Supplementary Material); interestingly, the two signals are both indicative for sulphur atoms covalently bonded to silver, but with two different hybridizations: the spin-orbit pair at lower BE (S2p3/2 = 161.05 eV) is indicative for S-Ag bonds with sp hybridized sulphur; the signal at higher BE (S2p3/2 = 162.09 eV) suggests S-Ag bonds with sp3 S atoms [78]. It is noteworthy that no physisorbed thiol moieties appear (R-SH S2p3/2 signals are expected around 163–164 eV BE); this finding is in excellent agreement with the hypothesis made by A Majzik et al. [61] based on ^1^H NMR studies and suggesting that in metal nanoparticles stabilized by mixed Cit and L-cys capping agents the L-cys molecules preferentially tend to directly bond the metal surface, inducing the most part of Cit molecules to form a shell around the first L-cys layer, in a “layer-by-layer”-like arrangement. It is noteworthy that XPS data do not allow excluding that some Cit molecules could intercalate between L-cys and directly interact with Ag atoms at the NP surface, as evidenced by IR spectroscopy results. The last signal reported in Figure 4d is the Hg4f spectrum. As evidenced in the figure, two spin-orbit pairs can be individuated; the first signal (Hf4f_7/2_ BE = 100.10 eV) can be associated with metallic Hg atoms [79]; the components at higher BE (Hg4f_7/2_ = 100.85 eV) are consistent with literature data reported for Hg^2+^ ions in oxides or coordination compounds [72].

The occurrence of a signal indicative for metallic Hg is in excellent agreement with the findings reported in [68], where for AgNPs stabilized by Cit molecules and interacting with Hg(II) ions, a direct interaction between Hg and Ag atoms at the nanoparticle surface was envisaged. To better understand the chemistry and geometry of Hg(II)–AgNPs interaction, XAS experiments at the Hg L_III_-edge are in programme. Actually, X-ray absorption experiments will allow to directly probe the local coordination chemistry of the metal ion, providing information complementary to the SR-XPS data and allowing for a precise description of the Hg coordination site.

### 3.4. AgNPs Ecosafety

**Figure 5:**
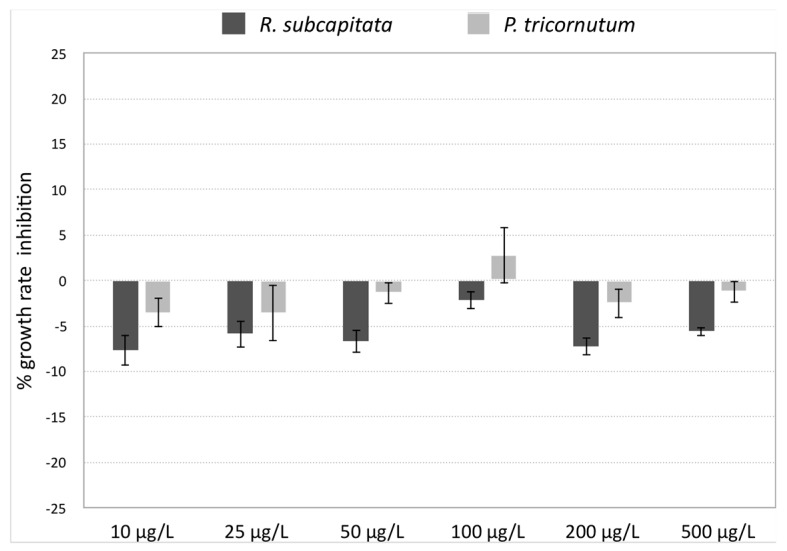
Percentage of growth rate inhibition compared to control of *R. subcapitata* and *P. tricornutum* exposed to AgNPs (10, 25, 50, 100, 200, 500 µg/L) for 72 h. Data are shown as mean ± standard deviation.

Algal toxicity tests with *R. subcapitata* and *P. tricornutum* exposed to AgNPs showed no inhibition of growth rate at all tested concentrations, as reported in Figure 5. Furthermore, algae showed a slight increase in growth rate compared to the control (negative growth rate inhibition), at almost all tested concentrations. Such increase, however, was not observed by positive control tests carried out with Cit and L-cys, which showed no effect on algal growth.

Reference toxicant (AgNO_3_) showed a 60% inhibition of growth rate compared to the control of *R. subcapitata* (freshwater), already at the lowest concentration tested (3.5 µg AgNO_3_/L), confirming the toxicity of Ag for microalgae. On the opposite, no effect on growth was observed for *P. tricornutum* (marine water) exposed in the same conditions, confirming the of drop in free Ag^+^ ions in solution due to complexation with Cl^−^ ions in seawater as measured by Gunsolus et al. [73].

The absence toxicity for our AgNPs confirms chemical analysis results showing insufficient release of Ag^+^ ions to exert a toxic effect on both microalgae (Table 1). Such findings further validate the hypothesis by which AgNP toxicity is closely bound to the release of Ag^+^ ions [44,46,47,54]. In fact, the dissolution of Ag^+^ from AgNPs, either pristine or coated (chitosan, lactate, polyvinylpyrrolidone, polyethelene glycol, gelatin, sodium dodecylbenzenesulfonate, Cit, dexpanthenol, carbonate), has been reported by many studies [44,45,46,47,48,52,54,80,81] and recognized to be linked to the observed toxicity to microalgae [44,46,47,54]. The covalently-bound L-cys coating of AgNPs might prevent the dissolution of Ag^+^ ions, since previous studies revealed that Cit coating was not able to avoid such release in aqueous media [44,46,47,73].

Molecules with a high reduced sulphur content are known to bind metal ions; L-cys, thanks to the presence of a thiol group, is able to act as a strong Ag^+^ ligand [82]. Thiol groups could act both either binding Ag^+^ ions, blocking their release in the media, or by excluding oxidizing agents to come in contact with the particles surface, preventing their dissolution and consequent release of Ag^+^ ions in the medium [83]. Gunsolus et al., [73] reported a significant reduction of Ag^+^ ions release and of bactericidal activity of citrate-AgNPs upon incubation with natural organic matter rich in thiol groups. Some studies observed a reduction in the toxic effect of Ag-releasing AgNPs after addition of L-cys in solution, probably as a consequence of L-cys complexation of free Ag^+^ ions [45,46,47].

However, information on L-cys effects on AgNPs stability and dissolution are conflicting. The presence of free L-cys in solution might also enhance Ag^+^ ions release from AgNPs [84]. In our study, however, the absence of toxicity to microalgae and low Ag levels in exposure media confirmed that Cit/L-cys coating prevents the release Ag^+^ ions from the NP. Similar results were obtained by Mu et al. [85] for graphene oxide (GO) nanosheets with covalently-bound L-cys, compared to pristine GO nanosheets. In terms of toxicity, for particles smaller than 20 nm an additional dissolution-independent effect seems to play a role [44,53]. However, despite being very small, AgNPs further confirm their integrity in terms of low Ag^+^ release. Since the main concern regarding the environmental application of AgNP is due to potential toxic effect to non-target aquatic species [44], our findings clearly showed the ecosafety of AgNPs and promote their use in the aquatic environment.

## 4. Conclusions

In this work AgNPs functionalized with hydrophilic capping agents, i.e., Cit and L-cys, were synthesized and characterized by means of UV-Vis, FT-IR, SR-XPS and NEXAFS spectroscopies, confirming the surface functionalization. Their nanodimensions were studied by DLS and TEM analysis, showing diameter less than 10 nm. AgNPs showed high selectivity and sensitivity for Hg^2+^ in water (concentration range:1–10 ppm) respect to 16 different metal ions investigated. The AgNPs-Hg^2+^ system was deeply investigated by means of DLS, ICP-MS, TEM and SR-XPS. Measurements of Ag concentration in fresh and marine aqueous media showed low Ag^+^ ions release, probably due to the good Cit/L-cys covering, also confirmed by SR-XPS data. Moreover, AgNPs ecosafety was confirmed by ecotoxicity tests which showed no effects on algal growth of both freshwater *R. subcapitata* and marine *P. tricornutum* algae in the range of tested concentrations (10–500 mg/L). Our results further support the hypothesis that the Cit/L-cys coating of AgNPs prevent dissolution of Ag^+^ ions in both fresh and saltwater. These results open new ways for AgNPs sensing applications in environmental tests on more complex biological systems, up to tests on real environmental aquatic scenarios.

## Figures and Tables

**Table 1:** Ag^+^ concentrations (expressed as µg/L) in freshwater and marine waters with algal medium solution (CTRL), as well as algal medium solutions with AgNPs (500 µg/L) and with AgNO_3_ (7 µg/L).

	TG 201 (Freshwater)		F/2 (Marine Water)	
	0 h	72 h	0 h	72 h
CTRL	0.36 ± 0.06	0.26 ± 0.08	0.27 ± 0.01	0.26 ± 0.02
AgNPs	0.19 ± 0.02	0.4 ± 0.06	0.15 ± 0.03	0.37 ± 0.03
AgNO_3_	4.42 ± 0.09	4.79 ± 0.18	4.37 ± 0.08	5.32 ± 0.22

**Table 2:** Comparison between dimension and limit of detection (LOD) of some metal NPs used as a colorimetric sensor for the detection of Hg^2+^.

Metal NPs Functionalization	NPs Diameter (nm)	Hg^2+^ Detection Limit (LOD) (M)	References
Citrate/Lcysteine-AgNPs	5 ± 2	3.0 × 10^−6^ (0.6 ppm)	This work
Tween 20-AuNPs	-	1.0 × 10^−7^	[8]
L-cys-AuNPs	-	1.0 × 10^−7^	[10]
*Acanthe phylum bracteatum* AgNPs	29–68	2.2 × 10^−6^	[40,74]
L-cysteine AgNPs	10	1.0 × 10^−8^	[75]
Glutamine/Histidine AgNPs	5.5 ± 1.0	9.0 × 10^−7^	[76]
Glutathione AgNPs	3.9 ± 0.6	>1.0 × 10^−7^	[77]

## Supplementary Materials

The following are available online at https://www.mdpi.com/2079-4991/9/10/1353/s1, Figure S1. TEM images of AgNPs; Figure S2. L-Cys FTIR spectrum; Figure S3. Uv-Vis spectra of of AgNPs in presence of 2.5 ppm of metal ions (As^3+^, As^5+^, Ca^2+^, Co^2+^, Mg^2+^, Nd^3+^, Ni^2+^, Zn^2+^, Pb^2+^) in water; all references curves (AgNPs alone) are in black line, while red curves represent the interaction between AgNPs and metal ions; Table S1. Table XPS. BE, FWHM, Atomic Ratio values and proposed assignments for all measured core-level signals title.
