# Supplementary material for: Bifunctionalized Silver Nanoparticles as Hg2+ Plasmonic Sensor in Water: Synthesis, Characterizations, and Ecosafety
Source: Nanomaterials (Basel). 2019 Sep 20;9(10):1353. doi: 10.3390/nano9101353 (PMC6835846; doi:10.3390/nano9101353)
Supplement: Supplementary file 1 [file nanomaterials-09-01353-s001.pdf]

Supplementary Material

Bifunctionalized Silver Nanoparticles as Hg^{2+} Plasmonic Sensor in Water: Synthesis, Characterizations, and Ecosafety

Paolo Prosposito [1,2], Luca Burratti [1], Arianna Bellingeri [3], Giuseppe Protano [3], Claudia Faleri [4], Ilaria Corsi [3], Chiara Battocchio [5], Giovanna Iucci [5], Luca Tortora [5,6], Valeria Secchi [5], Stefano Franchi [7] and Iole Venditti [5,*]

1 Department of Industrial Engineering and INSTM, University of Rome Tor Vergata, via del Politecnico 1, 00133 Rome, Italy; paolo.prosposito@uniroma2.it (P.P.); luca.burratti@uniroma2.it (L.B.)
2 Center for Regenerative Medicine, University of Rome Tor Vergata, Via Montpellier 1, 00133 Rome, Italy
3 Department of Physical, Earth and Environmental Sciences, University of Siena, Via Mattioli 4, 53100 Siena, Italy; arianna.bellingeri@student.unisi.it (A.B.); giuseppe.protano@unisi.it (G.P.); ilaria.corsi@unisi.it (I.C.)
4 Department of Life Sciences, via Mattioli 4, 53100 Siena, Italy; faleric@unisi.it
5 Department of Sciences, Roma Tre University of Rome, Via della Vasca Navale 79, 00146 Rome, Italy; chiara.battocchio@uniroma3.it (C.B.); giovanna.iucci@uniroma3.it (G.I.); luca.tortora@uniroma3.it (L.T.); valeria.secchi@uniroma3.it (V.S.)
6 Surface Analysis Laboratory INFN Roma Tre, via della Vasca Navale 84, 00146 Rome, Italy
7 Elettra-Sincrotrone Trieste S.c.p.A., Strada statale 14, km 163.5, 34149 Basovizza Trieste, Italy; stefano.franchi@elettra.eu
* Correspondence: iole.venditti@uniroma3.it; Tel.: +39-06-5733-3388

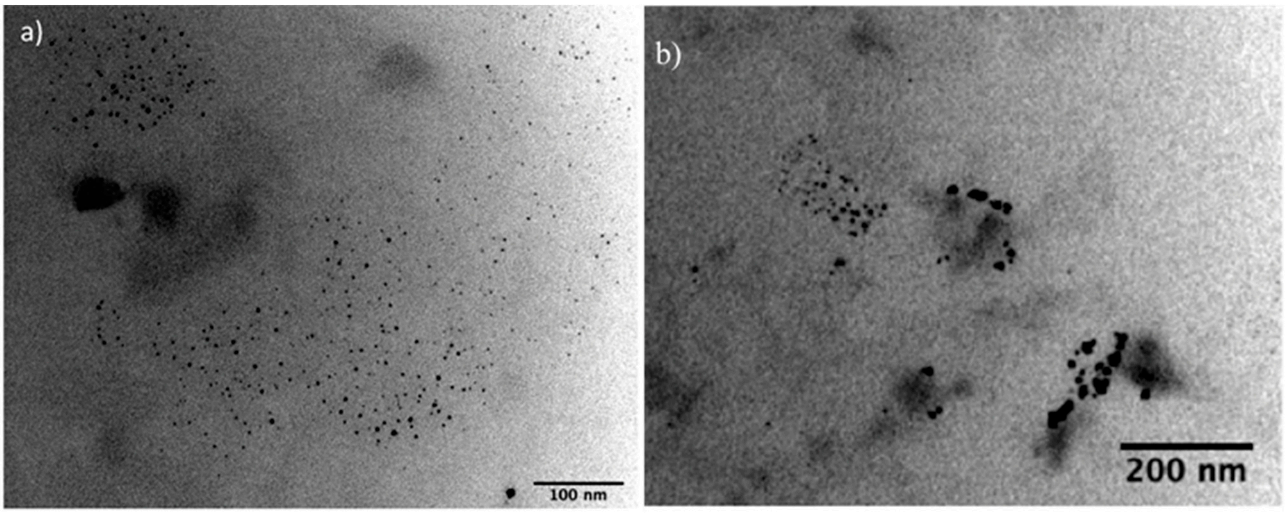

Figure S1: TEM images of bifunctionalized AgNPs at different magnifications.

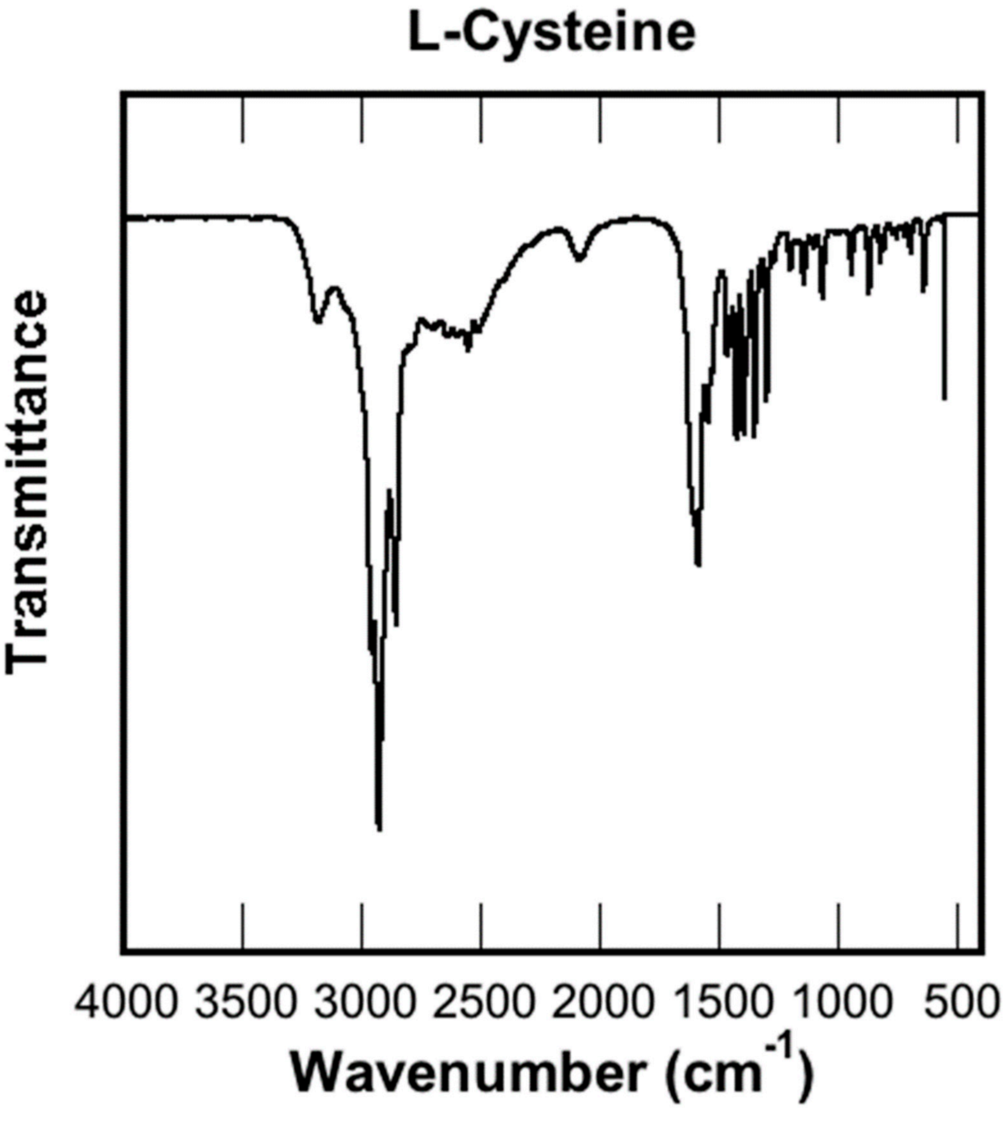

Figure S2: L-Cys FTIR spectrum.

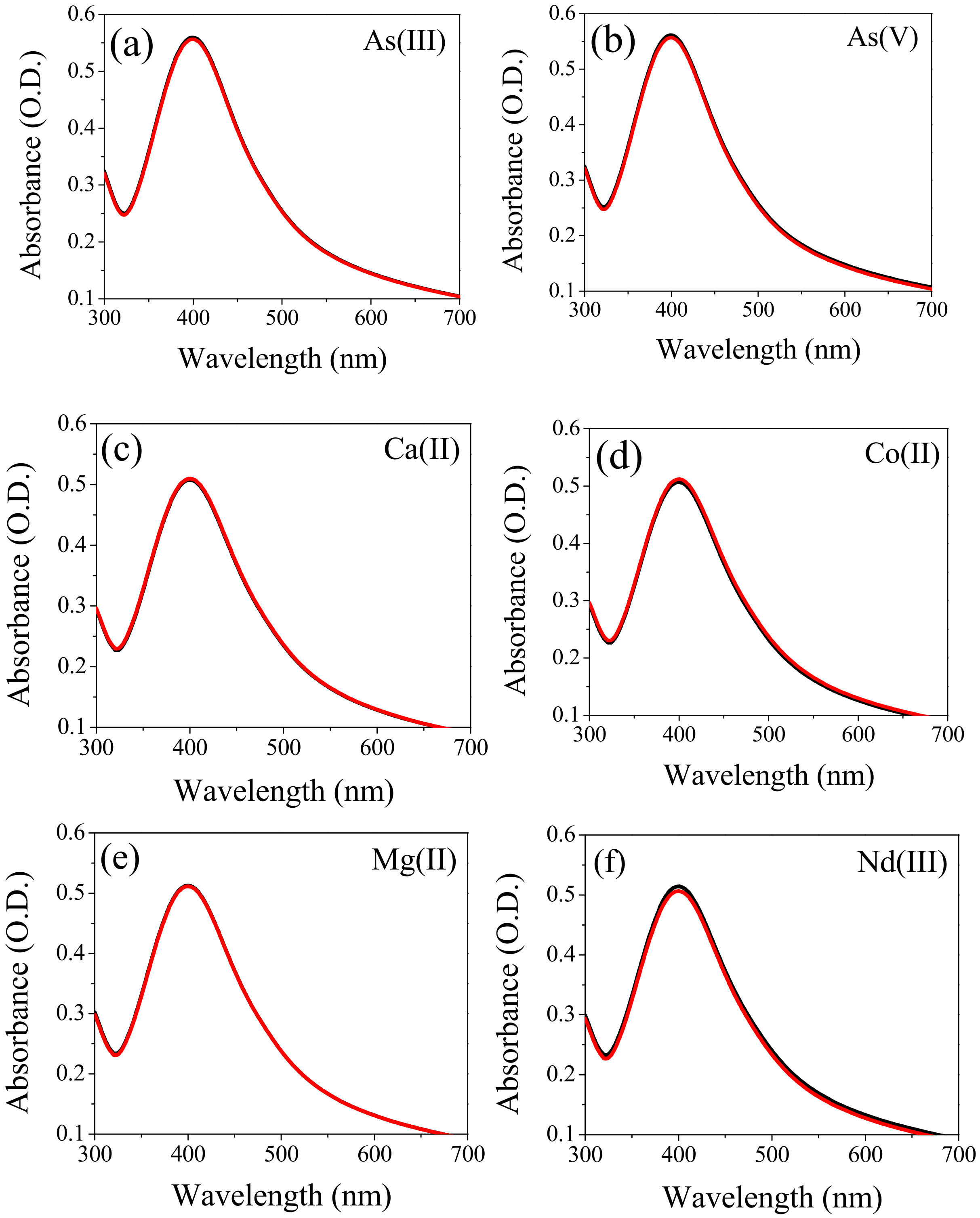
(a)
As(III)
(b)
As(V)
(c)
Ca(II)
(d)
Co(II)
(e)
Mg(II)
(f)
Nd(III)
Absorbance (O.D.)
Wavelength (nm)
0.1
0.2
0.3
0.4
0.5
0.6
300
400
500
600
700

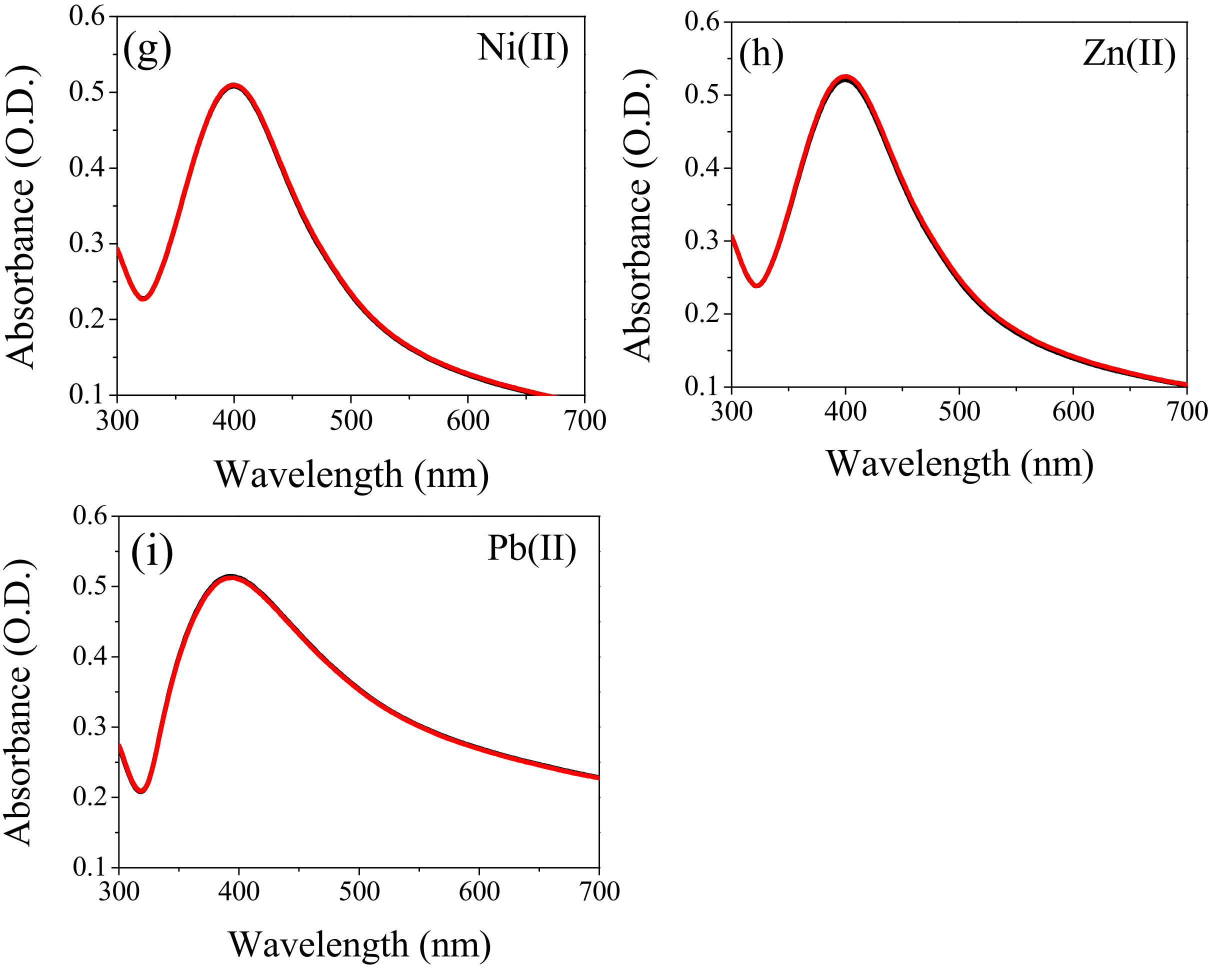

Figure S3. Uv-Vis spectra of of AgNPs in presence of 2.5 ppm of metal ions (As^{3+}, As^{5+}, Ca^{2+}, Co^{2+}, Mg^{2+}, Nd^{3+}, Ni^{2+}, Zn^{2+}, Pb^{2+}) in water; all references curves (AgNPs alone) are in black line, while red curves represent the interaction between AgNPs and metal ions.

Table S1. Table XPS: BE, FWHM, Atomic Ratio values and proposed assignments for all measured core-level signals.

Signal	BE (eV)	FWHM (eV)	Atomic Ratio (%)	Assignment
C1s	285.00	1.33	-	C-C
	286.46	1.33	47.9	C-S; C-N; C-O
	288.45	1.33	52.1	COOH
N1s	400.24	2.44	100.0	NR3
O1s	532.00	1.67	41.0	C=O
	533.00	1.67	59.0	O-H
	534.42	1.67	-	Physisorbed H_2O
S2p	161.05	0.95	54.4	S-Ag sp
	162.09	0.95	45.6	S-Ag sp^3
Ag3d	368.08	0.96	92.0	Ag(0)
	369.20	0.96	8.0	Ag(δ+)
Hg4f	100.10	0.93	83.7	Hg(0)
	100.85	0.93	16.3	Hg(2+)
